# Rigidity, chaos and integration: hemispheric interaction and individual differences in metaphor comprehension

**DOI:** 10.3389/fnhum.2014.00511

**Published:** 2014-07-14

**Authors:** Miriam Faust, Yoed N. Kenett

**Affiliations:** ^1^The Leslie and Susan Gonda (Goldschmied) Multidisciplinary Brain Research Center, Bar-Ilan UniversityRamat-Gan, Israel; ^2^Department of Psychology, Bar-Ilan UniversityRamat-Gan, Israel

**Keywords:** metaphors, creative language, cerebral hemispheres, network science, chaos, rigidity, integration

## Abstract

Neurotypical individuals cope flexibly with the full range of semantic relations expressed in human language, including metaphoric relations. This impressive semantic ability may be associated with distinct and flexible patterns of hemispheric interaction, including higher right hemisphere (RH) involvement for processing novel metaphors. However, this ability may be impaired in specific clinical conditions, such as Asperger syndrome (AS) and schizophrenia. The impaired semantic processing is accompanied by different patterns of hemispheric interaction during semantic processing, showing either reduced (in Asperger syndrome) or excessive (in schizophrenia) RH involvement. This paper interprets these individual differences using the terms *Rigidity, Chaos* and *Integration*, which describe patterns of semantic memory network states that either lead to semantic well-being or are disruptive of it. We argue that these semantic network states lie on a rigidity-chaos semantic continuum. We define these terms via network science terminology and provide network, cognitive and neural evidence to support our claim. This continuum includes left hemisphere (LH) hyper-rigid semantic memory state on one end (e.g., in persons with AS), and RH chaotic and over-flexible semantic memory state on the other end (e.g., in persons with schizophrenia). In between these two extremes lie different states of semantic memory structure which are related to individual differences in semantic creativity. We suggest that efficient semantic processing is achieved by semantic integration, a balance between semantic rigidity and semantic chaos. Such integration is achieved via intra-hemispheric communication. However, impairments to this well-balanced and integrated pattern of hemispheric interaction, e.g., when one hemisphere dominates the other, may lead to either semantic rigidity or semantic chaos, moving away from semantic integration and thus impairing the processing of metaphoric language.

## Introduction

Language is complex. Part of this complexity is the unique characteristic of human language that contains highly conventional as well as unconventional, more ambiguous and creative linguistic expressions such as novel metaphors (Faust, 2012; Mirous and Beeman, 2012). In the present paper we suggest that the ability of neurologically intact persons to cope flexibly with the full range of semantic relations expressed in language, including novel metaphoric relations, depends on the pattern of interaction between multiple brain networks in the two cerebral hemispheres during semantic processing. Specifically, we suggest that language is always a whole brain process and thus processing any type of language, including metaphors, requires integration between systemized, more rigid semantic processing associated with the left hemisphere (LH) and more flexible semantic processing associated with the right hemisphere (RH). However, when compared to more conventional types of language, processing novel metaphors may require relatively higher involvement of RH unique semantic coding.

The two cerebral hemispheres have been shown to code semantic information in different ways (for review see Mirous and Beeman, 2012). Much research indicates that RH mechanisms are highly sensitive to distant, unusual semantic relations, whereas LH mechanisms strongly focus on a few closely related word meanings while suppressing distant and unusual meanings (Brownell et al., 1983; Burgess and Simpson, 1988; Faust and Chiarello, 1998; Razoumnikova, 2000; Faust and Kahana, 2002; Bowden and Jung-Beeman, 2003; Faust and Lavidor, 2003; Mihov et al., 2010; Faust, 2012). The interaction between these two semantic systems can thus be described as lying on a *rigidity-chaos* semantic continuum. This continuum includes LH hyper-rigid and rule-based semantic processing on one extreme (e.g., in persons with Asperger syndrome (AS)), and RH chaotic and over-flexible semantic activation on the other extreme (e.g., in persons with schizophrenia). However, moving away from both LH *semantic rigidity* and RH *semantic chaos* leads to hemispheric well-balanced cooperation and to *semantic integration*. We suggest that this integration enables neurologically intact persons to process unconventional and ambiguous language, including both conventional and novel metaphoric expressions. Furthermore, we suggest that the level of semantic integration may be related to individual differences in creative ability.

Metaphors are considered to be part of the more creative aspects of language as they may require unusual semantic processing. Creativity is broadly defined as the creation of something which is both novel and useful, or appropriate (Mednick, 1962; Sternberg and Lubart, 1999; Runco and Jaeger, 2012). By this definition, a creative product is the combination of a flexible process, which allows generation of novel ideas, with a more systemized process which constrains novel concepts by their appropriateness (Nijstad et al., 2010). In line with this definition, creative language includes linguistic products which are both novel and appropriate, such as novel metaphors. Metaphors, including novel metaphoric expressions, are abundant in language (Lakoff and Johnson, 1980), as they allow efficient expressions of ideas that would otherwise be awkward to explain literally (Glucksberg, 2001). However, the use of metaphoric language requires the ability to activate a broader, more flexible set of semantic associations and combine weakly related concepts into a novel and appropriate linguistic product (i.e., sense creation, Bowdle and Gentner, 2005; Faust, 2012). Thus, metaphors are widely used in poetry, the ultimate expression of linguistic creativity, where with a few words, implicit and explicit emotions and associations from one context can powerfully be associated in a novel way with another, different context (Faust, 2012).

We have been working for the past two decades on processing of novel metaphors taken from poetry compared to conventional metaphors, literal expressions, and meaningless, unrelated word-pairs (reviewd in Faust, 2012). This research project used converging behavioral and neurocognitive techniques (accuracy and response times, split visual fields, Evoked Response Potentials (ERPs), Transcranial Magnetic Stimulation, functional Magnetic Resonance Imaging and Magnetoencephalography) to study neurotypical as well as clinical populations, such as persons with AS or schizophrenia (Faust, 2012; Gold and Faust, 2012; Zeev-Wolf et al., 2014).

This research project has consistently shown the contribution of the RH to novel metaphor processing in neurotypical persons and how deviation from a neurotypical state affects comprehension of novel metaphors (i.e., the processing of creative language): on one extreme, persons with AS exhibit rigidity of thought and have difficulties in processing novel conceptual combinations (novel metaphors) accompanied with reduced RH involvement (Gold and Faust, 2012); on the other extreme, persons with schizophrenia exhibiting loose associations, seem to have a different pattern of hemispheric involvement, including increased RH involvement. This different hemispheric pattern may result in the processing of unrelated word pairs as with meanings (Zeev-Wolf et al., 2014). Furthermore, the different patterns of hemispheric involvement in semantic processing characterizing persons with neurodevelopmental psychiatric disorders may be related to their documented deficits in the comprehension of nonliteral expressions while other language skills are relatively preserved (Martin and McDonald, 2004; Thoma and Daum, 2006; Rapp, 2012) [but see Gernsbacher and Pripas-Kapit (2012) for an alternative view on persons with AS].

The role of the RH in creative language is explained by the fine-coarse semantic processing model (FCT; Chiarello, 2003; Jung-Beeman, 2005) and is based on the notion that the RH uniquely activates and maintains a wide range of meanings and associations that enable the creation of novel conceptual combinations. This weak broad activation may better capture semantic relations which depend on the overlap of distantly related meanings. According to this theory, both hemispheres are involved in Bilateral semantic Access, Integration, and Selection (BAIS; Jung-Beeman, 2005), yet with a different processing role for each hemisphere. This difference implies different hemispheric mechanisms for metaphorical and literal language. When people comprehend literal language, the LH is strongly involved because the meaning is dominant, focal, and contextually relevant. However when people process metaphorical language, specifically novel metaphors; the RH plays a more important role because the figurative meaning of metaphors requires activations of loosely related concepts in a broader semantic field. The FCT has supporting neural evidence at both morphological and micro-anatomical levels (Mirous and Beeman, 2012). At the morphological level, there are a few distinct asymmetries between the LH and the RH, such as the LH having a larger temporal plane and a relatively higher ratio of gray to white matter and the RH having relatively more white matter and a higher degree of functional interconnectivity. At the micro-anatomical level, LH neurons have smaller input fields than RH neurons in language related brain areas. This difference in input fields may be related to more specific, fine, neural processing in the LH compared to less specific, coarser processing in the RH (Mirous and Beeman, 2012).

Several studies using functional Magnetic Resonance Imaging (fMRI) techniques to investigate hemispheric processing of metaphors have been conducted (Mashal et al., 2005; Schmidt and Seger, 2009; Yang et al., 2009; Diaz and Hogstrom, 2011; Diaz et al., 2011; Bohrn et al., 2012). Such studies show the involvement and contribution of the RH in processing novel metaphors and figurative language and how this involvement is affected by context, novelty, figurativeness, task difficulty and familiarity (Schmidt and Seger, 2009; Yang et al., 2009; Diaz and Hogstrom, 2011; Diaz et al., 2011). However, while much research has shown the role of the RH in metaphor processing, contradicting findings showing no RH role in metaphor processing and even LH dominance have also been reported (Rapp, 2012). Recent meta-analyses of several fMRI investigations of neural aspects of metaphor processing have yielded both LH and RH dominant activations (Rapp et al., 2012; Yang, 2014). Rapp et al. (2012) found more left-lateralized temporal network activation for processing non-literal language. Nevertheless, when the authors conducted subgroup analysis for different types of non-literal language types, they found more general bilateral and even more RH activated foci for non-salient, novel metaphor processing. Thus, this meta-analysis further strengthens the importance of bilateral hemispheric dynamics in the processing of non-literal language. Yang (2014) conducted an fMRI meta-analysis to investigate the role of the RH and the brain mechanisms involved in metaphor comprehension. This meta-analysis revealed that the RH is involved in metaphor comprehension and is influenced by conventionality, context and task demand. These factors might explain the contradicting evidence found in regard to the role of the RH in metaphor processing (Rapp, 2012). Furthermore, this meta-analysis related each of the three semantic processing components proposed by Jung-Beeman (2005) to neural activity, mainly the temporal lobe (medial temporal gyrus (MTG)/superior temporal gyrus (STG)) to semantic activation and integration and the frontal lobe (inferior frontal gyrus (IFG)) to semantic selection. Each component seems to involve bilateral brain regions activation, while RH regions perform coarser analysis than LH regions for the same process (Yang, 2014). Thus, while the role of the RH in metaphor processing is consistently shown, the importance of bilateral activation and hemispheric cooperation in creative and metaphoric language processing is becoming more and more apparent.

Evidence for bilateral activation in hemispheric processing of metaphors is slowly accumulating. Pobric et al. (2007) used repetitive Transcranial Magnetic Stimulation (rTMS) to study hemispheric involvement in semantic processing. They show that while RH interference only disrupted novel metaphor processing, LH interference disrupted literal and conventional metaphor processing, but facilitated novel metaphor processing (Pobric et al., 2007). These findings suggest that processing novel metaphoric relations requires dynamical, fine-tuned interaction between RH coarse and LH fine semantic processing mechanisms. Furthermore, it has been suggested that the corpus callosum mediates the processing of non-literal language such as metaphors, by the integration of relevant information between hemispheres (Thoma and Daum, 2006). Thoma and Daum (2006) show how persons suffering from agenesis of the corpus callosum (a congenital disease which results in complete or partial absence of the corpus callosum) are impaired in non-literal language processing.

These findings thus suggest a cognitive continuum which settles the contradicting evidence for hemispheric roles and interaction during metaphor processing and may provide a more general account for different patterns of neurocognitive processing of creative, including metaphoric, language exhibited by clinical and neurotypical persons (Gold et al., 2011; Gold and Faust, 2012; Zeev-Wolf et al., 2014). The continuum we propose here is a cognitive continuum of semantic processing states, ranging from rigidity to chaos. We borrow the notions of *rigidity*, *chaos* and *integration* from Siegel (2010) who uses these terms to describe psychological well-being and proposes a framework of *semantic* *well-being*. We will define the notions of semantic *rigidity*, *chaos* and *integration* and describe how network science allows for quantitative explorations of these notions. This is achieved by describing neural, computational and cognitive research in order to discuss the extreme states of semantic rigidity (via research on persons with AS) and semantic chaos (via research on persons with schizophrenia). Finally, we discuss semantic integration, which we have been recently exploring through the investigation of individual differences in semantic creativity.

## Semantic well-being—rigidity, chaos and integration

In presenting an integrative explanation for the psychological state of well-being, Siegel (2010) introduces the notions of rigidity, chaos and integration. As he sees it, emotional well-being is a state of integrative balance, leading to feelings of vitality and livelihood. The claim is that this balance is easily disrupted by deviation either towards too little arousal, a state of rigidity, or excessive arousal, a state of chaos (Siegel, 2010). This deviation from mental balance occurs frequently in mentally healthy persons, but extreme deviations can result in clinical conditions. Siegel claims that the key to mental balance is *integration*—linking together different elements from different system, which converges into a balanced synchrony, such as that of a singing choir in harmony (Siegel, 2010). Thus, emotional well-being is considered a balance of systems that integrate with each other and mental illness can be defined as a shift from a state of integration either to a rigid extreme or to a chaotic extreme. Searching for a theoretical framework to relate these notions, Siegel realized that network science allows quantitative definition and exploration of his theory of mental well-being (Siegel, 2010). In this paper, we used network science tools to quantify semantic rigidity, chaos and well-being and relate these processing modes to hemispheric involvement.

Network science is based on mathematical graph theory, providing quantitative methods to investigate complex systems as networks. A network is composed from nodes, which represent the basic unit of the system and links that represent the relations between them. This field has greatly advanced in the past few decades due to technological and quantitative theoretical advances. This rapid development has led to investigations of both properties (structural) and dynamics (such as emotional deviation from integration) of complex systems which can be represented as networks (reviewed in Baronchelli et al., 2013). Of the various network models developed in network science theory, the network model that has been widely used to examine complex systems is the Small World Network model (SWN; Milgram, 1967; Watts and Strogatz, 1998). SWN models have successfully described a wide range of sociological, technological, biological and economical networks (Boccaletti et al., 2006; Cohen and Havlin, 2010; Kenett et al., 2010; Newman, 2010). Two main characteristics of SWN are the networks clustering coefficient (CC) and its average shortest path length (ASPL). The CC refers to the probability that two neighbors (a neighbor is a node *j* that is connected through an edge to node *i*) of a node will themselves be neighbors. The ASPL refers to the average shortest amount of steps (nodes being traversed) needed to be taken between any pair of nodes. A SWN is characterized by having a large CC and a short ASPL.

At the cognitive level, application of network science tools is also developing, mainly to investigate complex systems of language and memory structure (Vitevitch, 2008; Borge-Holthoefer and Arenas, 2010; Chan and Vitevitch, 2010; Vitevitch et al., 2012, 2014; Baronchelli et al., 2013). In the linguistic domain, lexicons of different languages seem to display SWN characteristics, considered to be a fundamental principle in lexical organization (Steyvers and Tenenbaum, 2005; De-Deyne and Storms, 2008a,b; Borge-Holthoefer and Arenas, 2010; Kenett et al., 2011). Investigating the complexity of semantic knowledge with network science allows to uniquely examine fundamental questions such as the nature of semantic organization (what are the structural principles that characterize semantic knowledge?), process and performance (to what extent can human performance on semantic processing tasks be explained in terms of general processing in semantic memory network?) and typical and atypical semantic lexicon development (Steyvers and Tenenbaum, 2005; Beckage et al., 2011; Kenett et al., 2013). Network research in language is slowly shifting from an interest in investigating the structure of mental lexicons to investigating cognitive processes operating on these lexicon networks (reviewd in Borge-Holthoefer and Arenas, 2010). To date, no network model has been proposed to explain differences in creative language processing and specifically metaphor processing, both in healthy and clinical populations. Such a model must be able to provide a network based explanation and predictions to the differences found in a wide range of creative language processing modes, including metaphor comprehension.

A central concept in network theory is the random graph. A random graph is generally defined as any graph in which some parameters are fixed and some parameters are unconstrained, thus random (Newman, 2010). Random graphs were extensively studied by Erdös and Rényi, who defined a general model for a random graph (Erdös and Rényi, 1960). In their model, a graph consists of nodes (N) and a certain probability for the existence of a link between these two nodes (*L*), which is drawn via a Poisson distribution. This probability ranges from 0, where all of the nodes are disconnected from each other, to 1, where all of the nodes are connected to each other. Thus, having a fixed (N, *L*) results in a spectrum of different networks which vary in their connectivity patterns. It is important to note that the randomness of this model is based on its fixed parameters and that the model’s simplicity does not accurately model real world networks (Sporns, 2011). To better account for real world network properties (namely, high CC and low ASPL), Watts and Strogatz proposed a “small-world” random graph model (SW; Watts and Strogatz, 1998). Given a fixed number of nodes (N), fixed average degree (K; amount of nodes connected to a specific node *i*) and a probability parameter (p), a SW random graph is constructed in the following way: first, a regular network is constructed with N nodes connected to K neighbors. Next, every edge between a pair of nodes is rewired with a probability of p. Rewiring is defined as changing a link from connecting node *i* and node *j*, to connecting node *i* and node *k*. The rewiring process reshapes the structure of the random network such that it better represents real world networks (as described above). Such random graph models provide a quantitative framework to study different structural organizations of complex systems with a fixed number of nodes, such as brain networks or mental lexicons. In this sense, it is possible to examine how a varying degree of connectivity, which is brought about by variation in *L* affects cognitive processing and predicts individual differences and atypical cognitive conditions.

**Figure 1:**
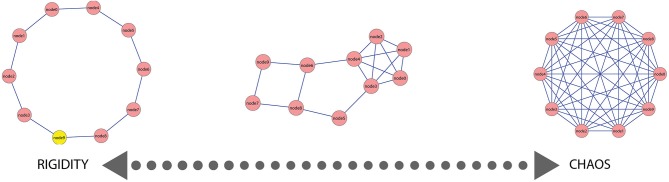
**A simulated example of the semantic continuum**. In this simulated example, 10 nodes are presented with varying connectivity patterns ranging from a rigid organization (extreme left) where each node is connected to only one other node, to a chaotic organization (extreme right) where each node is connected to all other nodes. In between lies a more integrated state (center) where part of the nodes are connected to few other nodes (rigidity) and a few nodes are connected to many other nodes.

We argue for a continuum of different mental lexicon states which constrains creative language including metaphor processing. This semantic continuum ranges from a mental lexicon state with extremely low connectivity (resulting in more ordered, rigid organization) to a mental lexicon state with extremely high connectivity (resulting in more random, chaotic organization) (Figure 1). In between lies a family of lexicon network states with varying connectivity structure, such as Barabasi-Albert or scale free networks (Cohen and Havlin, 2010). We suggest that a mental lexicon state which balances between these two extremes allows for an efficient processing of both conventional and creative language and also for the differentiation between these language types and meaningless linguistic expressions.

## Semantic rigidity

On one extreme of the semantic continuum are rigid networks. Such rigid networks are strongly ordered and minimally random, thus exhibiting a low CC and a high ASPL. Classical computational cognitive models which were proposed in the 1960’s to represent semantic memory are one such example (i.e., Collins and Quillian, 1969). Such models were structured tree-based representations and were criticized for their inability to account for flexible categorization (Rogers, 2008; Borge-Holthoefer and Arenas, 2010). Since metaphor processing requires the activation of wide, flexible associative networks (Faust, 2012), we propose that such rigid networks are inefficient in facilitating creative language processing. A distinctive example of how rigidity of thought disrupts creative language processing is found in persons with AS.

While persons with AS display relatively intact formal linguistic processing (syntax, morphology, phonology), they exhibit extensive difficulties in higher-level aspects of linguistic processing (Gold and Faust, 2012). Studies have shown that persons with AS show difficulty in understanding non-literal language, such as semantically ambiguous (Le Sourn-Bissaoui et al., 2011) and metaphoric (Gillberg and Gillberg, 1989; Gold et al., 2010; Gold and Faust, 2010, 2012) language.

Recent neural research in autism points to white matter deficiencies leading to disrupted connectivity, as suggested by the under-connectivity theory of autism (Just et al., 2004, 2012; Williams et al., 2013). This theory postulates that connectivity of inter-regional brain circuitry is disrupted, particularly affecting cognitive processes which demand integration of frontal-posterior brain interactions. This under-connectivity in autism has been shown in various cognitive processes, specifically in language comprehension (Just et al., 2004, 2012; Williams et al., 2013). McAlonan et al. (2009) investigated white matter deficits in children with AS and found that they have predominantly right sided white matter deficits, but also greater white matter volume than controls in LH language areas. Finally, Boger-Megiddo et al. (2006) have shown that children with autism have a disproportionately smaller corpus callosum volumes than typically developing controls.

These findings were further corroborated by functional and neuro-structural studies (Koshino et al., 2005; Nordahl et al., 2007) and converge with electrophysiological evidence showing disrupted RH for processing novel metaphors by persons with AS (Gold and Faust, 2010; Gold et al., 2010). Such electrophysiological research found no differences in the ERPs for processing novel metaphors compared to processing unrelated word pairs, in contrast to neurotypical controls. Thus, when persons with AS processed novel metaphoric and unrelated two word expressions, their N400 amplitudes did not differ, suggesting that they process novel, potentially meaningful semantic relations, as if they are meaningless. In addition, when persons with AS processed conventional and novel metaphors their N400 amplitudes were significantly more negative compared to neurotypical controls. No such difference was found in the N400 amplitude for processing literal or unrelated meanings in persons with AS. These findings suggest that for persons with AS, integration of novel metaphoric meanings is as difficult as the integration of unrelated, nonsensical meanings. The findings may thus provide electrophysiological evidence for the specific difficulties exhibited by persons with AS in processing creative language such as novel metaphors (Gold et al., 2010).

At the cognitive level, Gold and Faust (2012) have attempted to explain the difficulties in processing metaphoric language typically exhibited by persons with AS by extending the Empathizing-Systemizing theory proposed by Baron-Cohen (2009). This perspective argues that in the language domain, conventional language processing is rule-based and thus considered the more systemized part of semantic processing, which remains intact in persons with AS. Creative language processing, on the other-hand, involves some degree of semantic rule-violation strategies, such as the ability to violate conventional, dominant semantic relations and connect remote associations into a new and appropriate linguistic product. As such, the authors argue that creative language processing can be considered similar to the Empathic system, in the sense that is it much less rule-based, thus the processing of this type of language may be disrupted in persons with AS (Gold and Faust, 2012).

We have recently applied a network science research, investigating the structure of semantic memory of persons with AS compared to neurotypical controls (Kenett et al., under review). We show that the semantic memory structure of persons with AS is more compartmentalized than that of neurotypical controls—it breaks apart into smaller sub-parts. Community structure is extensively studied in network science, known as modularity (Newman, 2006; Fortunato, 2010; Meunier et al., 2010). The principle of modularity seems to be a fundamental principle of brain network organization and modularity disruption has been related to neurodegenerative diseases (Bullmore and Sporns, 2012). We claim that the hyper-modular structure of semantic memory in persons with AS is related to their rigidity of thought. We suggest that the hyper-modular mental lexicon network organization may hinder their ability to break apart from a specific module in the network and is thus related to their rigidity of thought.

In summary, we suggest that a systemized, highly conventional and relatively rigid processing is crucial for efficient processing of the more conventional, rule-based parts of language, associated with the LH. Nevertheless, extreme states of rigid semantic processing may disrupt the ability to process the more creative aspects of language, associated with higher RH involvement, as evident in persons with AS. Neural, behavioral and network research in persons with AS is beginning to converge to a possibly more coherent explanation of the difficulties such persons exhibit in processing the more creative types of language such as novel metaphors. Such difficulties may be related to an extremely rigid semantic system state, most likely as a result of neural under-connectivity which disrupts their ability for flexible semantic processing. Research on the effect of network rigidity on cognitive processing has only recently begun and requires much further research to better understand this effect (Arenas et al., 2012; Shai et al., 2014). We now turn to the other extreme end of the semantic state continuum—the chaotic state.

## Semantic chaos

On the other extreme of the semantic continuum lies chaotic state. In network science terms, chaotic networks can be defined as being random, or nearly random—high CC and very low ASPL, marking a network which is very highly connected and very little organized. Current neurocognitive studies which applied network science tools to investigate the developing brain have shown that brain network structures reorganize from an initial chaotic SWN state to a more structured network state (Boersma et al., 2011; Fan et al., 2011; Yap et al., 2011; de Bie et al., 2012; Smit et al., 2012; van Straaten and Stam, 2013). These studies provide neurophysiological evidence that complex brain networks start from a more chaotic, strongly small-worlded state, and slowly, as the brain matures, shift to a more structured state, while retaining SWN properties. Thus, in accordance with Siegel (2010) notion of well-being, the brain transgresses from a chaotic state to a more structured, balanced state. In fact, network research in neurodegenerative diseases show how such diseases alter healthy network states (van Straaten and Stam, 2013). Schizophrenia is a distinctive disease that results in altered neurocognitive network states.

Application of network science to EEG and fMRI data of individuals with schizophrenia has revealed loss of overall functional connectivity and small-world properties with increased network randomness (Micheloyannis et al., 2006; Liu et al., 2008; Rubinov et al., 2009; Lynall et al., 2010). Several fMRI studies have reported reduced clustering and reduced modularity in patients with schizophrenia (Liu et al., 2008; Alexander-Bloch et al., 2010), all supporting the network randomization theory of schizophrenia (Rubinov et al., 2009). Furthermore the severity of the disruption of the small-world structure of the brain seems to be related to the duration of the illness (Liu et al., 2008; Rubinov et al., 2009).

We have recently conducted behavioral and MEG research to investigate metaphor processing in persons with schizophrenia compared to neurotypical controls (Zeev-Wolf et al., 2014; Zeev-Wolf et al., in preparation). This research showed how the ability to differentiate between potentially meaningful, novel metaphoric expressions and meaningless word pairs may be deficient in persons with schizophrenia. Persons with schizophrenia appear to over-rely on coarse semantic coding, which may disrupt their ability to balance between finding new meanings to novel metaphors, on the one hand, and rejecting meaningless linguistic stimuli on the other hand. This was accompanied by a different pattern of hemispheric involvement during the processing of linguistic expressions. Specifically, we found a deficient pattern of RH excessive involvement for all types of expressions, mainly to novel metaphors, in persons with schizophrenia compared to neurotypical controls. Thus, at short c1 stimulus onset asynchrony (SOAS), while neurotypical persons exhibited a LH advantage for novel metaphor processing, persons with schizophrenia exhibited RH advantage. Furthermore, while neurotypical controls exhibited a LH advantage for processing literal and conventional metaphors, persons with schizophrenia exhibited a RH advantage (Zeev-Wolf et al., 2014). The MEG research provided further evidence for the unbalanced relations between hemispheres in processing novel metaphors. We found a general RH over-activation and unbalanced hemispheric activation during metaphor comprehension, as compared to neurotypical controls (Zeev-Wolf et al., in preparation).

At the cognitive level, network research investigating language and thought disorders in persons with schizophrenia is only initially developing (Mota et al., 2012; Voorspoels et al., 2014). Mota et al. (2012) used network tools to study speech acts produced by manic and schizophrenic patients, by creating speech graphs for each clinical population (Mota et al., 2012). This research shows how quantitative network measures can differentiate between persons with schizophrenia (by quantitatively accounting for the schizophrenic phenomena of “poverty of speech”), manic patients (by quantitatively accounting for the manic phenomena of “flight of speech”) and neurotypical controls, providing valuable clinical information not measured by classical clinical measurements. Further cognitive network research is required to better quantify the semantic memory of persons with schizophrenia and how it deviates from neurotypical controls, to better understand symptoms such as “loose associations”.

In summary, chaotic semantic network state allows for more flexible creative processing, associated with the RH. While a SWN state is a crucial aspect of neurocognitive structural and functional organization (Bullmore and Sporns, 2012; Baronchelli et al., 2013), an over chaotic SWN state may lead to cognitive deficiencies, as apparent in persons suffering from schizophrenia (Zeev-Wolf et al., 2014). As such, the semantic system must balance between rigidity, which may disrupt creative language processing and chaos, which may disrupt conventional, literal language processing. Furthermore, semantic chaos may also disrupt the processing of creative language, interfering with the appropriateness and relevance aspects of creative products. Each of these extremes thus affects the two components of the creative product—novelty (flexibility, chaos) and appropriateness (systemized, rigidity), respectively. This balance is achieved by integration.

## Semantic integration

To avoid the extreme states of either semantic rigidity or semantic chaos, we suggest that the neurocognitive system must strive for a balanced dynamics in its semantic processing. On the one hand, a highly-structured rule-based semantic system is advantageous to the cognitive system in regard to quickly retrieving the more conventional types of language such as literal meanings and highly conventional metaphoric expressions. This systematic, constraining semantic relation may thus offer a processing advantage for the rule-based semantic system of the LH. On the other hand, when the semantic relations between words comprising a linguistic expression are distant and unusual, such as in novel metaphors, the rule-based semantic system of the LH may require a complementary neural system that is able to cope with the potential rule violations created by non-conventional semantic combinations (Faust, 2012). These two systems must cooperate in a balanced manner, to achieve semantic, including metaphorical, well-being (Siegel, 2010) and to avoid extreme conditions where one system is dominant. Such unbalanced conditions can result in extreme rigidity, leading to an autistic-like state or extreme chaos, leading to a schizophrenic-like state (as reviewed above). Our notion of an interaction between a rule-based, more rigid, systemized linguistic LH system and a hyper-flexible, more chaotic, non-systemized linguistic RH system is supported by the fine coarse model, as described above (Mirous and Beeman, 2012). However, what might be the general neurocognitive basis for such a sub-division?

We have recently proposed a general account for the relations between two hemispheric systems that may support the creative process in different modalities (Kenett et al., under review). We suggest that creativity is not confined to the RH, but that it is a product of a dual system interaction in a given cognitive domain—a specialized neurocognitive system responsible for conventional processing and a non-specialized neurocognitive system responsible for unconventional processing. The interaction between these two systems allows for effective processing of both conventional and unconventional stimuli and may thus support creativity. We investigated our theory in a cognitive task in which the RH is the specialized system, namely face processing, in order to generalize the findings from language research to the visual domain. Face processing has been shown to be more typically processed by the RH (Yovel et al., 2008) thus allowing us to investigate our account. We show how conventional, natural faces are better processed by the specialized RH system, whereas unconventional faces are better processed by the non-specialized LH system (Kenett et al., under review). Furthermore, we show how only processing of unconventional faces presented to the non-specialized LH system is significantly positive related to creative ability (see Aziz-Zadeh et al., 2013 for supporting neural evidence). Thus, a well-balanced interaction between specialized and non-specialized neurocognitive systems seems to be critical for the efficient processing of all types of stimuli and mainly for coping with the less conventional, creative aspects of reality.

Our theory and findings are supported by the growing amount of research showing the importance of hemispheric communication for creativity (Razumnikova, 2007; Takeuchi et al., 2010; Zhao et al., 2014). Takeuchi et al. (2010) have found a significant positive correlation between the size of the corpus callosum and creative ability. The authors interpret their findings as supporting the idea that creativity is associated with “efficient integration of information” through integrated white matter pathways. In a follow up research, these authors conducted a resting state functional imaging research to investigate gray and white matter correlation with intelligence and creativity (Takeuchi et al., 2011). In regard to creativity, this research found a positive significant relation between white matter and creativity, further demonstrating the importance of white matter connectivity and creative ability. Recently, Zhao et al. (2014) conducted a functional connectivity research to examine hemispheric activation in verbal creativity. The authors report bilateral neural pathway activation with greater functional connectivity in the RH. It might be argued that this intra-hemispheric activation is required for the complex interplay between specialized and non-specialized systems in processing conventional and unconventional stimuli and even possibly conventional and unconventional features of a given stimulus.

From a network perspective, classical theory on creativity has directly related it to semantic (or associative) memory structure (Mednick, 1962; Runco and Jaeger, 2012). Mednick’s theory of individual differences in creativity proposes that high creative persons are characterized by “flat” (broader) instead of “steep” (few) association hierarchies (Mednick, 1962). Schilling (2005) proposed a SWN theory of creative insight problem solving, suggesting that insight is achieved via restructuring of semantic memory network. Rossman and Fink (2010) found that high creative persons give lower estimates of the distance between unrelated word pairs as compared to less creative persons, implying that high creative persons may have a wider, interconnected semantic network than low creative persons. Finally, we have recently conducted an empirical network research directly investigating Mednick’s notion of the difference between low and high creative persons (Kenett et al., 2014). We show how the semantic network of low creative persons is more rigid than that of high creative persons, thus providing empirical network support for Mednick’s theory (but see Benedek and Neubauer, 2013 for an alternative view). Thus, individual differences in creative ability may be constrained by semantic memory structure, which is in accord with our proposed semantic continuum. In line with this notion, as the semantic memory state is more rigid, thus it is “less creative”, till the point of a clinical state (persons with AS). On the contrary, as the semantic memory state is more chaotic, thus it is “more creative”, till the point of a clinical state (persons with schizophrenia).

In summary, semantic integration is crucial for semantic well-being and seems to be implemented by hemispheric communication between a specialized system and a non-specialized system. We propose that this explanation complements the fine-coarse semantic processing model and provides a comprehensive account for the contradicting role of the RH in metaphor processing (Faust, 2012; Rapp, 2012). Novel metaphor processing first requires sense retrieval of the conventional parts of the metaphor followed by a process of sense creation which links together the remote parts of the novel metaphor unto a new meaning (Bowdle and Gentner, 2005). Thus, activation of both hemispheres is required—each system contributing its unique processing and via efficient and flexible intra-hemispheric communication achieves semantic integration. This flexible interaction dynamics between the specialized and non-specialized systems may result in the ability to cope with the full range of semantic processing, including novel metaphor comprehension. However, deficient intra-hemispheric communication can result in the extreme states of the semantic continuum. We propose that individual differences in the relation between the LH specialized and RH non-specialized linguistic systems are related to differences in lexicon organization across the semantic continuum, as expressed in the difference between low creative versus high creative persons.

## Conclusions–the well-balanced semantic brain

We began this paper by stating that language is complex. We propose that language is a complex semantic system with varying types that require a delicate balance between the more rigid and the more chaotic aspects of semantic processing, striving for integration. This semantic integration is achieved by hemispheric communication and structural and functional neurocognitive connectivity. We propose a semantic continuum which ranges from extremely rigid to extremely chaotic mental lexicon organization. We argue that such a continuum can explain different modes of semantic processing in clinical (such as persons with AS or Schizophrenia) populations as well as individual differences in creative ability. We provide neural, behavioral and network science evidence, which converge to such a neurocognitive network continuum. Finally, we describe how different patterns of novel metaphor processing can be explained by such a continuum and how it reconciles between the contradicting evidence found in regard to the role of the RH and LH in metaphor processing.

Application of graph theory in neurocognitive research in the past two decades provided quantitative means to explore structure and dynamics of brain networks at all levels. So far, network science has been mainly used to investigate neural structural and functional networks, but such application is also growing in the investigation of the cognitive domain (Baronchelli et al., 2013). Network research at the neural level has identified two key principles of neural networks: functional segregation and integration (Bullmore and Sporns, 2012). In this paper we suggest that these two principles are at the basis of the cognitive task of metaphor processing: while a functional segregation of hemispheric systems operates on complementary types of stimuli, only through semantic integration is efficient metaphor processing achieved. We suggest that analyzing such different mental lexicon conditions which result in various communication, language and thought conditions can greatly contribute to the research of such conditions by bringing together seemingly unrelated findings and conditions and providing a theoretical framework to which they can be related.

A major direction for future research is to relate the systemized LH and the less systemized, more flexible RH semantic systems to the mental lexicon network. Mainly, are there dual parallel lexicon networks which allow efficient systemized and flexible processing? Or is there rather a general mental lexicon network which is somehow represented at the whole brain and operated differently by the systemized LH and flexible RH systems? While the growing mass of evidence of hemispheric communication during semantic processing (also supported by the FCT) seems to support the latter, future converging network and neurocognitive research is required to further investigate the matter. Recently, Caeyenberghs and Leemans (2014) conducted a network based fiber tractography analysis in order to reconstruct the LH and RH structural networks. These authors show how the LH is significantly more structured than the RH, whereas the RH is more small-worlded than the LH (see Caeyenberghs and Leemans, 2014 for a full description). Thus, these findings provide further neural support for our theory. Further research is needed to relate hemispheric network properties and the cognitive mental lexicon. Finally, further network research is required to better quantify our proposed semantic continuum. Mainly, how can a balanced integrated semantic state be quantified in network terms? Another such direction is the application of network science to study lexical organization in chaotic conditions, such as persons suffering from schizophrenia. Such research, which is currently lacking, can further strengthen our proposed semantic continuum and shed a unique light on this clinical condition. Finally, as brain organization at all levels adheres to a network organization, network science should be used to extend and develop neurocognitive models and theories. Such addition of a network layer to models and theories can help in restructuring current models; provide more general accounts and empirical predictions. The theory presented here is one such attempt.

## Conflict of interest statement

The authors declare that the research was conducted in the absence of any commercial or financial relationships that could be construed as a potential conflict of interest.
